# Measures and procedures utilized to determine the added value of microprocessor-controlled prosthetic knee joints: a systematic review

**DOI:** 10.1186/1471-2474-14-333

**Published:** 2013-11-27

**Authors:** Patrick JR Theeven, Bea Hemmen, Peter RG Brink, Rob JEM Smeets, Henk AM Seelen

**Affiliations:** 1 Adelante, Centre of Expertise in Rehabilitation and Audiology, Hoensbroek, The Netherlands; 2 Department of Rehabilitation Medicine, Maastricht University, Research School CAPHRI, Maastricht, The Netherlands; 3 Department of Traumatology, Maastricht University Medical Centre+, Maastricht, The Netherlands

**Keywords:** Rehabilitation, Functioning, Review, Classification, Amputation, Lower extremities, Microprocessor-controlled knee joint

## Abstract

**Background:**

The effectiveness of microprocessor-controlled prosthetic knee joints (MPKs) has been assessed using a variety of outcome measures in a variety of health and health-related domains. However, if the patient is to receive a prosthetic knee joint that enables him to function optimally in daily life, it is vital that the clinician has adequate information about the effects of that particular component on all aspects of persons’ functioning. Especially information concerning activities and participation is of high importance, as this component of functioning closely describes the person’s ability to function with the prosthesis in daily life. The present study aimed to review the outcome measures that have been utilized to assess the effects of microprocessor-controlled prosthetic knee joints (MPK), in comparison with mechanically controlled prosthetic knee joints, and aimed to classify these measures according to the components and categories of functioning defined by the International Classification of Functioning, Disability and Health (ICF). Subsequently, the gaps in the scientific evidence regarding the effectiveness of MPKs were determined.

**Methods:**

A systematic literature search in 6 databases (i.e. PubMed, CINAHL, Cochrane Library, Embase, Medline and PsychInfo) identified scientific studies that compared the effects of using MPKs with mechanically controlled prosthetic knee joints on persons’ functioning. The outcome measures that have been utilized in those studies were extracted and categorized according to the ICF framework. Also, a descriptive analysis regarding all studies has been performed.

**Results:**

A total of 37 studies and 72 outcome measures have been identified. The majority (67%) of the outcome measures that described the effects of using an MPK on persons’ actual performance with the prosthesis covered the ICF body functions component. Only 31% of the measures on persons’ actual performance investigated how an MPK may affect performance in daily life. Research also typically focused on young, fit and active persons.

**Conclusions:**

Scientifically valid evidence regarding the performance of persons with an MPK in everyday life is limited. Future research should specifically focus on activities and participation to increase the understanding of the possible functional added value of MPKs.

## Background

Prosthetic componentry has evolved considerably over the years, incorporating advanced materials and techniques into the modern-day prostheses. Presently, a multitude of prosthetic knee joints exists that, based on how these joints are controlled, can generally be divided into two groups, i.e. prosthetic knee joints with exclusively mechanical control properties, and microprocessor-controlled prosthetic knee joints (MPKs) [1]. Mechanical control of the knee joint during the swing phase of gait is achieved either by constant friction (spring loaded or elastic extension), hydraulic dampening, or pneumatic dampening. Mechanical control during stance phase is obtained by manual locking, a weight-activated friction brake, a polycentric axis, or by hydraulic dampening [1]. MPKs on the other hand, have a built-in microcomputer that continuously controls the flexion and extension resistance of the prosthetic knee during the stance phase and/or swing phase of each gait cycle. According to its manufacturers, the benefits of using an MPK, in comparison with a mechanically controlled prosthetic knee, are especially associated with the possibility to walk with varying walking speeds due to the adaptive swing phase control, and to walk with a higher level of safety due to the MPKs’ adaptive stance phase control. These features may allow persons with an amputation to ambulate with a walking pattern that closely resembles natural gait, which in turn may lead to, for instance, a reduction in metabolic cost and cognitive demand.

From the broad range of available components, rehabilitation professionals have to choose a specific prosthetic knee joint that enables the patient to function optimally with the prosthesis in daily life. This choice is a complicated and challenging task, because every patient is unique in terms of age, gender, body weight, type of amputation, physical condition, cognition, ambulation-related goals regarding activities of daily living, and societal roles (s)he wishes to engage in. It is crucial that a prosthetic knee joint is selected that best meets the patient’s characteristics and needs, in order for the patient to reach an optimal level of functioning in daily life. Patients’ level of functioning encompasses multiple health and health-related domains that are described in the framework provided by the International Classification of Functioning, Disability and Health (ICF) [2]. The ICF describes health and the health-related domains in two parts, i.e. “functioning and disability” and “contextual factors”. The part that covers functioning and disability is further categorised into the components “body functions”, “body structures” and “activities & participation”. The part describing contextual factors is further divided into “environmental factors” and “personal factors”. The ICF uses the term ‘functioning’ to encompass all body functions, body structures, activities and participation [2]. Additionally, amputees can be classified into subgroups based on their level of functioning. The Medicare Functional Classification Level system [3] (MFCL-0 through MFCL-4; or K0 through K4) is an example of such a classification. It classifies individuals based on their ability or potential ability to function with their prosthesis, as is indicated in Table 1. Such classification may aid clinicians in the selection of prosthetic knee joints, as the MFCL level of a patient is often associated with specific categories of prosthetic knee joints that are considered suitable for those patients [4].

**Table 1:** **Medicare functional classification level (MFCL)**[3]**descriptions**

**HCFA Modifier**		**MFCL description**
**K0**	MFCL-0	Does not have the ability or potential to ambulate or transfer safely with or without assistance and a prosthesis does not enhance quality of life or mobility.
**K1**	MFCL-1	Has the ability or potential to use a prosthesis for transfers or ambulation on level surfaces at fixed cadence. Typical of the limited and unlimited household ambulator.
**K2**	MFCL-2	Has the ability or potential for ambulation with the ability to traverse low-level environmental barriers such as curbs, stairs, or uneven surfaces. Typical of the limited community ambulator.
**K3**	MFCL-3	Has the ability or potential for ambulation with variable cadence. Typical of the community ambulator who has the ability to traverse most environmental barriers and may have vocational, therapeutic, or exercise activity that demands prosthetic utilization beyond simple locomotion.
**K4**	MFCL-4	Has the ability or potential for prosthetic ambulation that exceeds the basic ambulation skills, exhibiting high impact, stress, or energy levels, typical of the prosthetic demands of the child, active adult, or athlete.

HCFA = Health Care Financing Administration

Ever since the MPKs became available, a debate is ongoing as to what characteristics an amputee should have in order for an MPK to be a more suitable prosthetic solution than a mechanically controlled prosthetic knee joint to function optimally in daily life. Finding an answer to this issue is an important step in streamlining the current prescription guidelines and reimbursement policies regarding the MPKs. Various studies with diverse degrees of quality have been performed that evaluated the possible effects on persons’ level of functioning of using the more advanced MPKs in comparison with the mechanically controlled prosthetic knee joints. The effects of using an MPK have been assessed using a variety of outcome measures in a variety of health and health-related domains. However, if the patient is to receive a prosthetic knee joint that enables him to function optimally in daily life, it is vital that the clinician has adequate information about the effects of that particular prosthetic device on all ICF components that comprise persons’ level of functioning. Especially information concerning the ICF component “activities and participation” is of high importance, as that component closely describes the person’s ability to function with the prosthesis in daily life. Moreover, information is necessary that describes to what amputee subgroups (e.g. MFCL classes) those effects are applicable. A comprehensive overview of the scientific evidence concerning the differences in the effects of MPKs and mechanically controlled prosthetic knee joints on the functioning of amputees is therefore warranted. A first step in creating such an overview is to investigate how and for which components of amputees’ functioning those effects have been determined thus far.

The present review aims:

1) to identify the outcome measures that have been utilized to assess the effects of using an MPK in comparison with a mechanically controlled prosthesis in persons with a knee disarticulation or transfemoral amputation, 2) to classify and structure the outcome measures identified according to the ICF framework, 3) to evaluate the characteristics and quality of the studies that have been performed to assess the differences between MPKs and mechanically controlled prosthetic knee joints, 4) to determine possible gaps in the information that is available concerning the three ICF components ‘body functions’, ‘body structures, and ‘activities and participation’.

## Methods

### Search strategy

The databases PubMed, CINAHL, Cochrane Library, Embase, Medline and PsychInfo were searched on May 1, 2013. The search focused on studies that compared the use of any type of MPK to the use of any type of mechanically controlled prosthetic knee joint. The following default Boolean search strategy was used: “prosthe* AND knee AND (amput* OR disarticulation) AND (microprocessor OR active OR electronic* OR magnetorheologic* OR intelligent OR variable-damping OR computerized)”. The search was limited to articles written in English, Dutch, French, or German between January 1990 and April 2013. The first MPK became commercially available in 1993. In addition to the database search, the lists of references of all papers included were checked for eligible articles.

### Screening

The papers found in the literature search were independently screened by two researchers (PT and HS), based on title and abstract, and classified as ‘relevant’, ‘not relevant’, or ‘possibly relevant’. The two researchers resolved any disagreement regarding the classification of papers by assessing the full-text article and discussing on ultimate inclusion or exclusion. Subsequently, the full text of all papers, found to be eligible, was reviewed. Papers were included when they met the following inclusion criteria:

(1) Participants are persons with a transfemoral amputation or knee disarticulation;

(2) Persons’ performance using a mechanically controlled knee joint is contrasted to persons’ performance using an MPK;

Papers were excluded when one or both of the inclusion criteria were not met, when they focused on endoprosthetic knee joints, or when the primary outcome parameters focused on performance of the prosthetic knee joint and not on the performance of the person using the prosthesis.

### Classification of outcome measures identified

**Figure 1:**
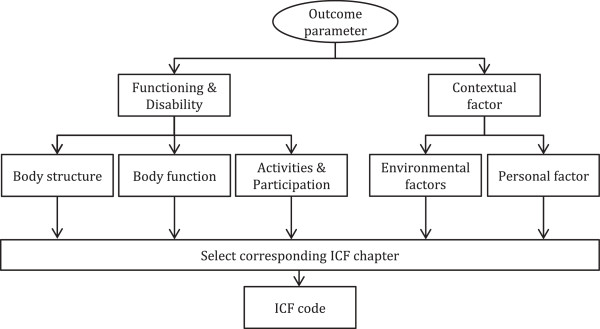
Decision tree utilized to assign an ICF code to the outcome parameters identified.

Main outcome parameters were identified in the selected studies. Each parameter was then categorised using the decision tree shown in Figure 1. This decision tree represents the main structure of the ICF classification [2]. It was used as a tool to identify the particular aspect of functioning associated with each outcome parameter. Subsequently, an ICF code was assigned following the general coding guidelines provided by the ICF (ICF annex 2 [2]) to further indicate the specific body structure, body function, activity, participation, personal factor or environmental factor the outcome parameter focused on.

Also, a distinction was made between parameters that describe persons’ actual performance and persons’ self-perceived performance. Actual performance was defined as the objectively detectable level of functioning of the amputee in a given domain at a given moment. Perceived performance was defined as the level of functioning subjectively experienced by the amputee in a given domain at a given moment (self-report).

Furthermore, it was specified for what particular purpose each body function, body structure, activity and participation, environmental factor, or personal factor was measured. The main topics of the papers included in the present review are described by the authors as: ‘quality of walking’, ‘energy cost’, ‘cognitive effort’, ‘safety’, ‘activity level’, ‘activities other than walking’, ‘prosthetic comfort’ and ‘other (e.g. health)’. In order to improve the clinical relevance of the present review these domains were used to classify the outcome of the literature search.

### Descriptive analysis of studies included

A descriptive analysis of the studies included in the present review was performed. Information is provided about the characteristics of the participants included in the studies (i.e. age, aetiology of amputation, reported functional level), and the characteristics of the studies included (sample size, study design, methodological quality). The methodological quality was assessed using the van Tulder’s quality assessment system. This scale scores the internal validity (maximum 11 points), the descriptive criteria (maximum 6 points) and the statistical criteria (maximum 2 points) [5,6].

## Results

**Figure 2:**
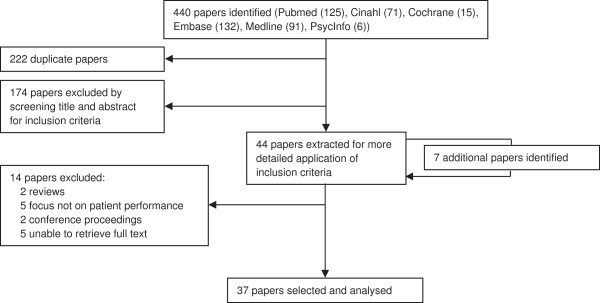
Schematic representation of article selection.

Figure 2 represents a schematic overview of the selection process of the studies. A total of 37 articles, in which the use of mechanically controlled prosthetic knee joints was contrasted to the use of MPKs, were included in the final analysis.

A comprehensive overview of the characteristics of the papers included is presented in Additional file 1: Table S1. Papers are sorted in ascending order of publication year.

### Classification of outcome measures identified

A detailed description of the main outcome parameters extracted from the articles is shown in Table 2. A total of 72 different outcome parameters were identified. Thirty-eight parameters described participants’ actual performance, 30 parameters described participants’ self-perceived performance, and 4 parameters assessed both actual performance and perceived performance (indicated in Table 2 with “a-p”).

**Table 2:** Overview of outcome parameters identified in studies included in review

		**Outcome parameter**	**ICF code**[2]	**Actual or perceived performance**	**Quality of walking**	**Energy cost**	**Cognitive effort**	**Safety**	**Activity level**	**Activities other than walking**	**Prosthetic comfort**	**Other (e.g. health)**
**Functioning & disability**	**ICF component** **“Body”** *(comprising “Body functions” and “Body structures”)*	walking speed [7,14,16-18,20,21,24,30,35],[39]	b770	a	●					●		
		(a)symmetry [7,16,18,29,36,40,41]	b770	a	●							
		stance phase duration [15,35,39,41]	b770	a	●					●		
		joint angles [15,17,18,21,39]	b770	a	●					●		
		joint moments [15,17,18,21,26]	b770	a	●							
		joint power [16,20]	b770	a	●							
		step time [17,41]	b770	a	●							
		step length [17,21]	b770	a	●							
		single-support time [17,39,41]	b770	a	●					●		
		double-support time [17,41]	b770	a	●							
		jerk [17]	b770	a	●							
		cadence [14,18,24,35,39]	b770	a	●					●		
		ground reaction force [21,41]	b770	a	●							
		impulse [41]	b770	a	●							
		stride length [14,24,35,39]	b770	a	●					●		
		knee extension instant [35]	b770	a	●							
		latency period [35]	b770	a	●							
		muscular activity pattern [17,39]	b760	a	●					●		
		walking distance [14,25]	b455	a	●							
		dynamic stability [39]	b755/b235	p	●							
		O_2_ consumption rate [7-9,13,14,16-18,20,27,31]	b455	a		●						
		heart rate [13,14,18,27]	b455	a		●						
		physiological cost index [24,29]	b455	a		●						
		physical activity-related energy expenditure [31]	b455	a		●						
		effort to walk [7,10,18,31]	b469	p		●						
		ability to divide attention [18,22]	b140	p			●					
		concentration [25,34]	b140	p			●					
		whole body sway [11]	b755	a			●					
		joint angles/moments (safety potential) [33]	b770	a				●				
		joint angles (gait pattern variability) [18]	b770	a				●				
		confidence (walking [7,25,32,34]/standing [7]/balance [28,39,43])	b126	p/p/p				●				
		perceived safety [18,32]	b126	p				●				
		moment of heel off [39]	b770	a						●		
		postural stability [26]	b755/b235	a						●		
		load on non-affected leg [18]	b780	p								●
		integration in body scheme [18]	b180	p								●
		body image [23]	b180	p								●
	**ICF** **component** **“Activities & Participation”**	quality [10,16,18]/ ability [10,12,18,29,32] of walking	d450	a-p/p	●							
		learning to walk [10]	d155	p			●					
		stumble frequency [25,29,30,34]		p				●				
		fall frequency [25,29,30,34,43]		p				●				
		activity duration [19,42]		a					●			
		activity level [42]		a					●			
		number of daily bouts of activity [42]		a					●			
		daily step frequency [19,25]	d450	a					●			
		quality [25,34]/ability [10,18,29,30]/effort [7] of stairs negotiation	d455	a/p/p						●		
		quality [25,34]/ability [10,18,29]/effort [7] of hill negotiation	d450	a/p/p						●		
		ability [10,25,30,34]/effort [7] to negotiate uneven terrain	d450	a-p/p						●		
		quality of obstacle negotiation [27]	d450	a						●		
		duration [37]/symmetry [37] of sitting down/standing up	d410	a/a						●		
		basic functional mobility [25,34,39,43]	d4*	a						●		
		ability to perform daily life activities [38]	d4*/d649	a-p						●		
		satisfaction/performance [25,30,31,34,39,42]		p								●
		general health/QoL [25,27,34]		p								●
		participation in work/leisure activities [43]	d850/d920	p								●
**Contextual factors**	*ICF Environmental & Personal factors*	amount of prosthetic use [39]	e1151	p	●						●	
		use of walking aids [18,24,39]	e115	a-p	●							
		swing phase control [12]	e1151	p							●	
		knee dynamics [12]	e1151	p							●	
		mechanical reliability [10]	e115	p							●	
		socket fit [30]	e1151	p							●	
		prosthesis attributes [29,32]	e1151	p							●	
		physical effects of prosthesis [32]	e1151	p							●	

* = applies to multiple paragraphs in chapter 4 (d410-d499); a = actual performance; p = perceived performance; ICF = International Classification of Functioning, Disability and Health.

Classification according to the ICF framework demonstrated that 54% of all outcome parameters assessed the effects of using an MPK at the ICF component ‘body functions’, none of the outcome parameters focused on the ICF component ‘body structures’, and 35% at ICF component ‘activities and participation’. An additional 11% of the outcome parameters were classified as environmental factors. In 7 cases, no specific ICF code could be ascribed to the parameter, because of the wide interpretability of those parameters.

Furthermore, 67% of all parameters that described persons’ actual performance targeted the ICF body functions component and 31% the ICF activities and participation component. For parameters that describe persons’ perceived performance, 32% measured the ICF component body functions and 44% the ICF component activities and participation.

In thirty-two studies [7-11,13-18,20-36,39-41,43] (i.e. 87% of all studies), one or more parameters were utilized that focussed on ICF body functions to assess the differences in performance between an MPK and a mechanically controlled prosthesis. Fifty-one percent of all parameters assessing the ICF body functions component, divided over 18 studies [7,14-18,20,21,24-26,29,30,35],[36,39-41] (i.e. 49% of all studies), targeted persons’ quality of walking with the prosthesis. These parameters typically encompass spatiotemporal parameters, kinematic, and kinetic parameters, measured with standardised gait analysis. An additional 13% of all parameters assessing the ICF body functions component, divided over 14 studies [7-10,13,14,16-18,20,24,27,29],[31] (38% of all studies), focused on the energy cost of walking with the prosthesis. Furthermore, 8% of the parameters assessing ICF body functions focused on cognitive effort (5 studies [11,18,22,25,34]), 15% on safety-related parameters (9 studies [7,18,25,28,32-34,39,43]), 23% on activities other than walking (i.e. standing and ramp descent) (2 studies [26,39]), and 8% on other parameters (2 studies [18,23]).

Seventeen studies [7,10,12,16,18,19,25,27],[29,30,32,34,37-39,42,43] measured one or more parameters assessing the ICF component activities and participation to determine the level of performance with the prosthesis. The majority (57%) of all parameters assessing ICF activities and participation described activities other than walking on level ground. These activities included a variety of daily life activities, including negotiation of stairs, hills, uneven terrain, and obstacles. Also, sitting down and standing up was assessed, as well as a variety of basic mobility tasks.

### Descriptive analysis of studies included

The MPKs investigated in the studies were the C-leg (Otto Bock HealthCare, Duderstadt, Germany)(n = 27), the C-leg Compact (Otto Bock HealthCare, Duderstadt, Germany)(n = 3), Intelligent Prosthesis (Chas A. Blatchford & Sons Ltd, Basingstoke, UK)(n = 7), Rheo knee (Össur, Reykjavik, Iceland)(n = 1), Adaptive knee (Chas. A. Blatchford & Sons Ltd, Basingstoke, UK)(n = 1), Power knee (Össur, Reykjavik, Iceland)(n = 1), and a newly developed polycentric MPK prototype (n = 1). Accommodation time for the MPK ranged between 30 minutes to 44 months.

In all studies a cumulative total of 810 participants was investigated, not taking into account the possibility that a same subset of participants may have been described in multiple papers. The mean age of participants was 44.9 years (sd 9.7 years; range = 15-85 years). The most common aetiology of amputation for the participants in the studies were trauma, congenital causes and malignancy (n = 28 studies) [7-13,15-19,23,25,26,28,30-38,40-42]. Participants with an amputation due to peripheral vascular diseases were included in 12 studies [18,25,26,30-32,34,37,38,40],[42,43]. In 26 [7-13,16-19,21,22,25-28,30-34,36,37],[40,43] of the 37 studies the participants were described as active and fit individuals. One hundred and two participants (i.e. 12.6% of total number of subjects) with a reported lower functional level (≤MFCL-2) were included, divided over eight studies [18,25,30,34,37-39,42]. Five of those studies [18,34,38,39,42] specifically investigated the effects of MPKs in amputees classified as MFCL-2. In eight studies [14,15,20,23,24,29,35,41] participants’ functional level was not clearly specified.

The mean sample size of participants for all studies included was 21.9 (sd 59.3; range 1–368). The sample size of the study of Berry et al [32] (n = 368) may be considered a far outlier. The mean sample size without this study was 12.3 (sd 9.9; range 1–42). In 35 [7-34,36,38-43] of the 37 studies, a within-subject design was used to test the differences between mechanically controlled prosthetic knees and MPKs. Two studies [35,37] compared the performance of a cohort of amputees using mechanically controlled prostheses with one or two cohorts of amputees using a particular type of MPK.

The methodological quality of all studies included, indicated by the mean total van Tulder score, was 9.2 (sd 1.7; range 6–12) out of a maximum score of 19. Mean score on the internal validity of the publications was 4.5 (sd 0.8) out of 11. The mean score on descriptive criteria was 3.2 (sd 0.9) out of 6, and the mean score for the statistical criteria was 1.5 (sd 0.5) out of 2. When using the cut-off value suggested by van Tulder (50% of the maximum score), the methodological quality was acceptable in 22 [13,15,17,19-23,25-27,30-34,36-40,42] of the 37 studies.

## Discussion

The main aim of the present review was to provide insight in how the effects of using an MPK, in comparison with a mechanically controlled knee joint, have been determined thus far. To this end, an overview of the outcome measures that have been utilized in comparative studies to describe the effects of an MPK on patients’ were identified from the literature and subsequently structured according to the ICF framework to provide a comprehensive overview of the components within the health and health-related domains that have been assessed. In addition, a descriptive evaluation of the study characteristics, the participant characteristics, and the overall quality of the studies performed, was conducted. The findings indicated that a majority (67 %) of all outcome measures, utilized to assess persons’ actual performance, targeted the ICF component ‘body functions’, whereas only few measures assessed the effects of using an MPK at activity and participation (31%). The outcome measures utilized to evaluate persons’ perceived performance with an MPK also typically described the effects on body functions and activities and participation. Thus, the scientific knowledge that is currently available regarding the effects of using an MPK on persons’ functional abilities is limited. Additional information is necessary about how the use of an MPK may affect persons’ actual ability to perform activities in everyday life and how using an MPK may influence people’s participation in society.

The majority of the evidence that is available regarding the effectiveness of using an MPK is primarily based on measures covering the ICF body functions component. Such information is valuable to understand the principles behind how an MPK may contribute to optimizing persons’ performance with a prosthesis. However, one of the main reasons to provide an above knee amputee with a prosthesis featuring a specific prosthetic knee joint is to enable that person to reach an optimal level of functioning in daily life. Yet, only few studies have investigated the effects of an MPK on activities and participation component of the ICF. Information about this ICF component is more closely related to the problems experienced by amputees in daily life in comparison with measures concerning body functions. For instance, persons with an above-knee amputation are not necessarily interested in whether they consume less millilitres of oxygen per kilogram per meter walked on a treadmill at a constant speed. Instead, it is more important for them to know whether they are (more) able to perform their activities of everyday life, or be involved in life situations when using their prosthesis.

The outcome measures utilized to measure functioning in the activities and participation domain were predominantly based on parameters on perceived performance. As this entails subjective information about persons’ performance, it is more sensitive to bias (e.g. response or recall bias) compared with objective measurements. Persons may give answers that are influenced by their own expectations, or because they want to please the researcher. Also, subjective measures are more sensitive to changes in the participants’ frame of reference, i.e. their perception may change over time, for instance, because they have put a certain condition or state into perspective. Nevertheless, it is important that, in addition to information concerning persons’ actual performance level, the level of participants’ perceived performance is also included in the process of decision making for a prosthetic knee, as persons’ level of (dis)satisfaction with the prosthesis is strongly related to the level of use of the prosthesis [44,45]. Unfortunately, in only 8 of the 31 studies, identified in the current review, parameters were assessed by measuring a combination of both actual and self-perceived performance. The assessment of a combination of both actual and perceived performance measures is strongly recommended for future prosthetic research.

To objectively measure a person’s actual performance regarding ICF activity and participation is difficult, due to the fact that very few (validated) measuring tools are available, that have been designed specifically to be used in persons with an amputation of a lower extremity [46]. Nevertheless, several original tools that objectively measure activities were found in the present review. Theeven et al [38] have developed and used a test, the Assessment of Daily Activities Performance in Transfemoral amputees test (ADAPT), that includes 17 common daily activities that have to be performed by the person being measured. With ADAPT it is possible to reliably and objectively measure amputees’ functional abilities to perform daily activities [47]. Hafner et al [25,34] used three measures that aim to assess actual performance at ICF activities and participation. The Stair Assessment Index (SAI) [48] and Hill Assessment Index (HAI) [49], that measure persons’ overall ability and quality of performance during stairs and hill negotiation, and a distracted walking test, that assesses the level of cognitive load during walking. During the latter test, persons walked outdoor around a busy city block while talking to a researcher on a cell phone. Participants had to repeat series of numbers back to the researcher in reversed order. Datta et al [16] used an original video observation scale to evaluate persons’ overall gait quality. Also, Seymour et al [27] developed a standardised walking obstacle course to determine persons’ ability to negotiate obstacles. Although little information is available about the psychometric properties of the aforementioned measures, they seem ecologically valid. Tools that aim to measure participation are typically self-report measures. This is inherent to the definition of participation, as it involves social interaction, which is impossible to measure under standardised conditions.

Although this review focused on how the effects of an MPK were assessed, it should also be noted that the majority (≈70%) of all studies that investigated the effectiveness of using an MPK included relatively young, fit, and active persons, with an amputation due to trauma (i.e. a classification of at least MFCL-3). In contrast, only twelve studies [18,25,26,30-32,34,37,38,40],[42,43] included persons with an amputation due to peripheral vascular disease and in only five studies [18,34,38,39,42] the effects of an MPK were specifically evaluated in amputees classified as MFCL-2. This does not seem to be representative of the total amputee population. The prevalence numbers in, for instance, the United States and the Netherlands, indicate that an estimated 42% and 90% respectively, of the lower limb amputees are over 65 years old [50], and around 79% and 94% respectively, of the amputations are due to peripheral vascular disease [50,51]. The research attention concerning the evaluation of MPKs seems to be more focused on the high profile, well-performing subpopulation. However, it is important to recognise that the ageing of the general population and the increasing prevalence of obesity and cardiovascular disease, are likely to cause the relative number of older persons with an amputation for vascular problems to grow. Persons with a lower functional level might also benefit from using a prosthesis featuring an MPK [18,24,34,38,39,42]. However, additional research is necessary to further increase our knowledge available about the effects of MPKs regarding this subpopulation of prosthesis users.

The average quality of the studies identified in the review was moderate to low. In general, the effects of the MPKs were investigated in a small research population, leading to statistical power problems and affecting the generalizability of the results reported. Also, large variation exists in the length of the accommodation time for the MPK (range 30 minutes to 44 months). Intuitively, a longer accommodation time would result in a better performance on the tests. English et al [52] reported that at least one week of functional walking with the prosthesis is necessary for clinical decision making on the suitability of a knee joint, but that three weeks is recommended for research purposes. This recommendation, however, is based on the findings in one subject. To date, no consensus exists on a suitable accommodation period. Additionally, the mean Van Tulder score for all studies incorporated in the review (9.1 out of 19), further underlines the moderate quality. According to Van Tulder [5,6] the methodological quality of a study is considered adequate with a score of at least 9.5. Especially, the internal validity is scored very low (4.4 out of 11), which may be associated with the lack of blinding in all studies. Blinding of the participants is practically impossible to accomplish, as the different features of the MPKs have to be explained to enable subjects to use the MPK to its full potential. It should also be noted that the Van Tulder’s quality assessment system is typically designed to rate randomised controlled trials (RCT) or controlled clinical trials (CCT). The majority of the studies included in the present review were neither. This may have led to an underestimation of the quality of the papers in the review.

### Recommendations for future research

The majority of the comparative studies have objectively investigated the effects of an MPK versus a mechanically controlled prosthesis on walking (quality, physical effort, and cognitive effort). Little information is available about other aspects of functioning with a prosthesis. Next to the investigations on negotiating stairs, slopes, and obstacles, only one study [38] assessed persons’ actual ability to perform activities of daily living (ADL). Research should focus more on whether the effects measured in a human movement lab also apply to situations in persons’ everyday life, i.e. investigate the functional added value of using a prosthetic knee joint.

Future research should focus on the development of tools that are able to objectively measure actual performance in persons with an above-knee amputation. More specifically, tools that measure activity and participation are necessary to further increase our understanding about the possible effects of using an MPK on persons’ functioning. Moreover, a combination with parameters on self-perceived performance is warranted.

Prosthesis research should focus more on the effects of using an MPK in persons with a lower functional level (e.g. amputees classified as MFCL-2). Given their limited physical capacity, these persons might not benefit from using the adaptive swing phase control that should enable them to walk at varying speeds. However, they may possibly benefit from the higher levels of stability of the knee due to the continuous adaptive stance phase control.

## Conclusions

Much research has been done on the effects of MPKs. However, the information available provides insight into the effects of using an MPK on only a limited number of health and health-related domains. The effects have been predominantly investigated from the perspective of the body (ICF body functions), and so, objective information and scientifically valid evidence regarding the performance of persons with an MPK in everyday life is still fairly limited. Research should therefore specifically focus on activity and participation, rather than body functions and body structures.

Also, the information about the effects of MPKs on persons’ functioning cannot be generalised for the entire population of persons with an above-knee amputation, as testing of MPKs is almost only performed in healthy, fit, and active persons, who are relatively young. Future research should therefore also aim at the older, less active persons with an amputation to complement the information available so far.

## Abbreviations

ADAPT: Assessment of daily activities performance in transfemoral amputees; ADL: Activities of daily living; CCT: Controlled clinical trial; ESK: Endolite stabilised knee; HAI: Hill assessment index; HCFA: Health care financing administration; ICF: International classification of functioning, disability and health; IP: Intelligent prosthesis; l/r: Left/right; MFCL: Medicare functional classification level; MPK: Microprocessor-controlled prosthetic knee joint; PEQ: Prosthesis evaluation questionnaire; PSPC: Pneumatic swing phase control; PVD: Peripheral vascular disease; QoL: Quality of life; RCT: Randomised controlled trial; SAI: Stair assessment index; sd: Standard deviation; SF-36: 36-item short-form health survey; VCO2: Rate of carbon dioxide production; VO2: Rate of oxygen consumption; VT: Van Tulder score.

## Competing interest

The authors declare that they have no competing interests.

## Authors’ contributions

PT and HS performed the data acquisition. PT, BH, HS, and RS participated in the study design and data analysis. PT, BH, and HS have been involved in the initial drafting of the manuscript. All authors, i.e. PT, BH, HS, PB, and RS, participated in critically revising the manuscript and in the preparation of the manuscript. All authors have read and approved the final manuscript.

## Pre-publication history

The pre-publication history for this paper can be accessed here:

http://www.biomedcentral.com/1471-2474/14/333/prepub

## Supplementary Material

Additional file 1: Table S1 Overview of study characteristics, outcome parameters identified, and results on main outcome parameters. Click here for file
