# Supplementary material for: Measures and procedures utilized to determine the added value of microprocessor-controlled prosthetic knee joints: a systematic review
Source: BMC Musculoskelet Disord. 2013 Nov 27;14:333. doi: 10.1186/1471-2474-14-333 (PMC3890588; doi:10.1186/1471-2474-14-333)
Supplement: Additional file 1: Table S1 — Overview of study characteristics, outcome parameters identified, and results on main outcome parameters. [file 1471-2474-14-333-S1.docx]

**Table 2** Overview of study characteristics, outcome parameters identified, and results on main outcome parameters

| Reference | Sample size (n) | Age ± sd (range) | Study design | Study quality (VT) | Functional classification level reported | Type of MPK | Type of mech. knee | Accommodation time with MPK | Measuring tools | Main outcome measures |
| --- | --- | --- | --- | --- | --- | --- | --- | --- | --- | --- |
| Kirker 1996 [7] | 14 | questionnaire  (n = 14) ? ± ? (18-66)  walk tests  (n = 6) 36.5 ± 6.2 (29-44) | within-subject | 7 | - trauma, congenital;  - good health;  - walk at different   speeds | IP | PSPC | 4-7 months | - original questionnaire;  - 100m walk test;  - expired gas analysis during   treadmill walking;  - video recording of treadmill   walking | questionnaire |
|  |  |  |  |  |  |  |  |  |  | - effort to walk at varying speeds; |
|  |  |  |  |  |  |  |  |  |  | - effort to walk outdoors or at work; |
|  |  |  |  |  |  |  |  |  |  | - effort to negotiate stairs; |
|  |  |  |  |  |  |  |  |  |  | - effort to negotiate slopes; |
|  |  |  |  |  |  |  |  |  |  | - confidence in knee when walking; |
|  |  |  |  |  |  |  |  |  |  | - confidence in knee when standing; |
|  |  |  |  |  |  |  |  |  |  | - knee preference; |
|  |  |  |  |  |  |  |  |  |  |  |
|  |  |  |  |  |  |  |  |  |  | walk tests |
|  |  |  |  |  |  |  |  |  |  | - walking speed (self-selected, fast, slow); |
|  |  |  |  |  |  |  |  |  |  | - VO_2_; |
|  |  |  |  |  |  |  |  |  |  | - symmetry in step length |
| Taylor 1996 [8] | 1 | 33 | within-subject | 7 | - trauma;  - active | IP | Mauch SNS; PSPC | ≥5 weeks | - expired gas analysis during   treadmill walking | - VO_2_;  - VCO_2_ |
| Buckley 1997 [9] | 3 | 48.3 ± 10.1 (39-59) | within-subject | 8 | - trauma;  - fit;  - active | IP | PSPC | ? | - expired gas analysis during   treadmill walking | - VO_2_ |
| Datta 1998 [10] | 22 | 39.9 ± ? (25-76) | within-subject | 7 | - trauma;  - osteomyelitis,   malignancy;  - fit;  - generally fairly   active | IP | ESK with PSPC | 7-41 months | - original questionnaire | - ability to walk at varying speeds; |
|  |  |  |  |  |  |  |  |  |  | - distance able to walk; |
|  |  |  |  |  |  |  |  |  |  | - ability to negotiate stairs; |
|  |  |  |  |  |  |  |  |  |  | - ability to negotiate slopes; |
|  |  |  |  |  |  |  |  |  |  | - ability to walk on uneven underground; |
|  |  |  |  |  |  |  |  |  |  | - effort to walk; |
|  |  |  |  |  |  |  |  |  |  | - walking style; |
|  |  |  |  |  |  |  |  |  |  | - mechanical reliability; |
|  |  |  |  |  |  |  |  |  |  | - learning to walk; |
|  |  |  |  |  |  |  |  |  |  | - comments by others on walking style |
| Heller 2000 [11] | 10 | 38 ± ? (23-46) | within-subject | 8 | - trauma, congenital;  - generally fit;  - active | IP | ESK | ≥6 weeks | - video-based motion analysis during   treadmill walking at constantly   varying walking speeds | - mean forehead marker displacement velocity of   walking with and without cognitive dual task |
| Stinus 2000 [12] | 15 | 39.6 ± ? (27-70) | within-subject | 7 | - trauma; | C-leg | 3C1; 3R49; 3R72; 3R80; ESK; Teh lin pneumatic; Mauch SNS; ESK IP | 6-14 months | - original questionnaire | - perceived stance phase safety; |
|  |  |  |  |  | - 11 subjects very   active |  |  |  |  | - perceived swing phase control; |
|  |  |  |  |  |  |  |  |  |  | - perceived dynamics of the knee; |
|  |  |  |  |  |  |  |  |  |  | - recommendation prosthesis to others; |
|  |  |  |  |  |  |  |  |  |  | - continuous duration able to walk; |
|  |  |  |  |  |  |  |  |  |  | - continuous distance able to walk |
| Schmalz 2002 [13] | 6 | 37 ± 9 (27-53) | within-subject | 10 | - trauma;  - able to walk 5 km   daily | C-leg | 3C1 | ? | - expired gas analysis during   treadmill walking;  - heart rate monitor | - VO_2_;  - heart rate |
| Perry 2004 [14] | 1 | ≈30 | within-subject | 6 | - infection;  - bilateral   amputation; | C-leg | Mauch SNS | ≈8 months | - expired gas analysis on outdoor   track;  - electrocardiography;  - insoles with compression closing   switches | - stride length; |
|  |  |  |  |  |  |  |  |  |  | - cadence; |
|  |  |  |  |  |  |  |  |  |  | - walking distance in 20 minutes; |
|  |  |  |  |  |  |  |  |  |  | - walking speed during 20 minutes; |
|  |  |  |  |  |  |  |  |  |  | - VO_2_; |
|  |  |  |  |  |  |  |  |  |  | - heart rate |
| Yokogushi 2004 [15] | 3 | 28 ± 1.5 (?-?) | within-subject | 10 | - trauma | micro-processor swing phase (4-bar) | 3R60 | 30 minutes | - video-based motion analysis;  - 2 force platforms | variation with different cadence in: |
|  |  |  |  |  |  |  |  |  |  | - stance phase duration (% of gait cycle); |
|  |  |  |  |  |  |  |  |  |  | - peak knee flexion angle (stance + swing); |
|  |  |  |  |  |  |  |  |  |  | - peak hip flexion angle; |
|  |  |  |  |  |  |  |  |  |  | - peak hip extension moment |
| Datta 2005 [16] | 10 | 38 ± ? (23-46) | within-subject | 8 | - trauma, congenital;  - generally fit;  - reasonably high   level of activity | IP | ESK | ≥6 weeks | - expired gas analysis;  - video observation scale (VAS);  - video-based motion analysis | - VO_2_; |
|  |  |  |  |  |  |  |  |  |  | - overall quality of gait; |
|  |  |  |  |  |  |  |  |  |  | - walking speed; |
|  |  |  |  |  |  |  |  |  |  | - symmetry in stance time; |
|  |  |  |  |  |  |  |  |  |  | - symmetry in step distance |
| Johansson 2005 [17] | 8 | 44.3 ± ? (29-54) | within-subject | 11 | - trauma, infection,   congenital, cancer;  - ≥ MFCL-3 | C-leg;  Rheo knee | Mauch SNS | 10 hours/ knee | - portable expired gas analysis on   indoor track;  - video-based motion analysis;  - 2 force plates;  - electromyography;  - accelerometers; | - VO_2_; |
|  |  |  |  |  |  |  |  |  |  | - walking speed; |
|  |  |  |  |  |  |  |  |  |  | - step time; |
|  |  |  |  |  |  |  |  |  |  | - step length; |
|  |  |  |  |  |  |  |  |  |  | - single support time; |
|  |  |  |  |  |  |  |  |  |  | - double support time; |
|  |  |  |  |  |  |  |  |  |  | - joint angles (hip, knee, ankle); |
|  |  |  |  |  |  |  |  |  |  | - joint moments (hip, knee, ankle); |
|  |  |  |  |  |  |  |  |  |  | - joint power (hip, knee, ankle); |
|  |  |  |  |  |  |  |  |  |  | - muscular activity pattern (M. Gluteus Max +   Med); |
|  |  |  |  |  |  |  |  |  |  | - jerk (smoothness of motion) |
| Wetz 2005 [18] | 25 | 45 ± ? (16-77) | within-subject | 7 | - trauma, tumour,   malformation,   infection, PVD;  - MFCL-2;  - MFCL-3;  - MFCL-4 | C-leg | Mauch SNS;  ESK;  Total knee;  Teh Lin;  3R80;  3R60;  KP3;  3R49;  3R40 | ? | - electronic walkway system;  - video-based motion analysis;  - 2 force plates;  - portable expired gas analysis;  - original questionnaire | - symmetry in step length; |
|  |  |  |  |  |  |  |  |  |  | - symmetry in stance phase duration; |
|  |  |  |  |  |  |  |  |  |  | - cadence; |
|  |  |  |  |  |  |  |  |  |  | - variability in gait pattern; |
|  |  |  |  |  |  |  |  |  |  | - walking speed; |
|  |  |  |  |  |  |  |  |  |  | - joint angles (hip and knee); |
|  |  |  |  |  |  |  |  |  |  | - joint moments (hip and knee); |
|  |  |  |  |  |  |  |  |  |  | - VO_2_; |
|  |  |  |  |  |  |  |  |  |  | - heart rate; |
|  |  |  |  |  |  |  |  |  |  | - perceived safety; |
|  |  |  |  |  |  |  |  |  |  | - load on non-amputated leg; |
|  |  |  |  |  |  |  |  |  |  | - integration into body schema; |
|  |  |  |  |  |  |  |  |  |  | - divided attention; |
|  |  |  |  |  |  |  |  |  |  | - ability to walk with varying speeds; |
|  |  |  |  |  |  |  |  |  |  | - reduction of effort of walking; |
|  |  |  |  |  |  |  |  |  |  | - quality of gait; |
|  |  |  |  |  |  |  |  |  |  | - use of walking aids |
| Klute 2006 [19] | 5 | 48 ± 12 (?-?) | within-subject | 11 | - trauma, tumour;  - ambulate without   walking aids | C-leg | Mauch SNS | 3 months | - activity monitor | - daily step frequency;  - activity duration |
| Orendurff 2006 [20] | 8 | 48.5 ± 10.2 (?-?) | within-subject | 10 | ? | C-leg | Mauch SNS | 3 months | - portable expired gas analysis on   indoor track;  - velocity-recording cart | - VO_2_;  - walking speed (self-selected) |
| Segal 2006 [21] | 8 | 47 ± 13 (28-60) | within-subject | 10 | - ambulate without   walking aids on   level ground, stairs   and inclines | C-leg | Mauch SNS | 3 months | - video-based motion analysis;  - force plate | - walking speed (self-selected); |
|  |  |  |  |  |  |  |  |  |  | - step length; |
|  |  |  |  |  |  |  |  |  |  | - peak knee flexion angle (stance); |
|  |  |  |  |  |  |  |  |  |  | - knee flexion angle at opposite heel strike; |
|  |  |  |  |  |  |  |  |  |  | - peak knee flexion angle (swing); |
|  |  |  |  |  |  |  |  |  |  | - peak knee flexion moment; |
|  |  |  |  |  |  |  |  |  |  | - peak coronal knee moment of intact limb; |
|  |  |  |  |  |  |  |  |  |  | - peak sagittal-plane joint power (hip, knee,   ankle); |
|  |  |  |  |  |  |  |  |  |  | - maximal vertical ground reaction force (vGRF) |
| Williams 2006 [22] | 8 | 48.5 ± 10.2 (29-61) | within-subject | 12 | - able to walk 3   flights of stairs and   30m on an incline   without walking   aids | C-leg | Mauch SNS | 3 months | - serial subtraction test;  - controlled oral word  - association test;  - category test;  - original questionnaire  - prosthetic cognitive burden scale | - number of errors; |
|  |  |  |  |  |  |  |  |  |  | - total number of unique words; |
|  |  |  |  |  |  |  |  |  |  | - total number of unique words; |
|  |  |  |  |  |  |  |  |  |  | - attention focused on walking; |
|  |  |  |  |  |  |  |  |  |  | - attention focused on thinking of words; |
|  |  |  |  |  |  |  |  |  |  | - attention focused on other things.; |
|  |  |  |  |  |  |  |  |  |  | - general subjective cognitive burden |
| Bunce 2007 [23] | 42 | 45.5 ± 12.5 (?-?) | within-subject | 10 | - trauma (n = 30) | C-leg | ? | 6 months | - amputee body image scale;  - interview | - body appearance; |
|  |  |  |  |  |  |  |  |  |  | - body function; |
|  |  |  |  |  |  |  |  |  |  | - effective distress; |
|  |  |  |  |  |  |  |  |  |  | - behavioural avoidance in social situations |
| Chin 2007 [24] | 2 | 78 ± 4.2 (75-81) | within-subject | 7 | ? | IP | 3R15;  3R40 | 12 weeks training | - heart rate monitor | - physiological cost index; |
|  |  |  |  |  |  |  |  |  |  | - step length; |
|  |  |  |  |  |  |  |  |  |  | - cadence; |
|  |  |  |  |  |  |  |  |  |  | - stride length; |
|  |  |  |  |  |  |  |  |  |  | - walking speed; |
|  |  |  |  |  |  |  |  |  |  | - use of walking aids |
| Hafner 2007 [25] | 17 | 49.1 ± 16.4 (21-77) | within-subject | 10 | - trauma,   dysfunction,   malignancy,   infection, PVD;  - MFCL-2;  - MFCL-3 | C-leg | Seattle Mark V; Mauch SNS; Teh Lin; Total knee; CaTech; Otto Bock Active; Ultimate; 3R60; | 1-32 weeks | - activity monitor;  - amputee mobility predictor;  - 36-item short-form health survey   (SF-36);  - Stair Assessment Index (SAI);  - Hill Assessment Index (HAI);  - 73.2m outdoor obstacle course;  - distracted walking test;  - Prosthesis Evaluation   Questionnaire (PEQ);  - original 14-question PEQ   addendum | - activity level (step frequency + estimated daily   distance travelled); |
|  |  |  |  |  |  |  |  |  |  | - basic functional mobility; |
|  |  |  |  |  |  |  |  |  |  | - self-reported general health (QoL); |
|  |  |  |  |  |  |  |  |  |  | - ability to negotiate stairs (functional   independence and technique); |
|  |  |  |  |  |  |  |  |  |  | - ability to negotiate slopes (functional   independence, technique, step length, speed); |
|  |  |  |  |  |  |  |  |  |  | - ability to negotiate uneven terrain (speed); |
|  |  |  |  |  |  |  |  |  |  | - cognitive demand of walking (test speed and   accuracy); |
|  |  |  |  |  |  |  |  |  |  | - self-assessed satisfaction; |
|  |  |  |  |  |  |  |  |  |  | - self-assessed performance; |
|  |  |  |  |  |  |  |  |  |  | - confidence; |
|  |  |  |  |  |  |  |  |  |  | - concentration; |
|  |  |  |  |  |  |  |  |  |  | - number of stumbles; |
|  |  |  |  |  |  |  |  |  |  | - number of falls |
| Kaufman 2007 [26] | 15 | 42 ± 9 (26-57) | within-subject | 10 | - trauma, tumour,   PVD, congenital;  - ≥ MFCL-3 | C-leg | Mauch SNS or equivalent | 10-39 weeks | - video-based motion analysis;  - 2 force plates;  - sensory organisation test (SOT) | - peak knee extensor moment during stance;  - postural stability (equilibrium score) |
| Seymour 2007 [27] | 13 | 46 ± 13 (30-75) | within-subject | 11 | - non-vascular;  - MFCL-4 | C-leg | Mauch SNS; 3R80; Total knee; 3R90; CaTech; Seattle Fusion | 2-44 months | - expired gas analysis during   treadmill walking;  - 12.2m standardised walking   obstacle course;  - 36-item short-form health survey   (SF-36) | - VO_2_; |
|  |  |  |  |  |  |  |  |  |  | - heart rate; |
|  |  |  |  |  |  |  |  |  |  | - ability to negotiate obstacles (speed, number of   steps, number of step-offs (errors), number of   stumbles); |
|  |  |  |  |  |  |  |  |  |  | - self-reported general health (QoL) |
| Stevens 2007 [28] | 1 | 30 | within-subject | 8 | - trauma;  - participates in   running and cycling | C-leg | 3R80 | 9 days | - Activities-specific Balance   Confidence scale (ABC) | - balance confidence score |
| Jepson 2008 [29] | 5 | 41.2 ± ? (28.8-55.7) | within-subject | 8 | ? | Adaptive | CaTech | 8 weeks | - video-based motion analysis;  - force plate;  - physiological cost index (PCI);  - original questionnaire | - symmetry in step length; |
|  |  |  |  |  |  |  |  |  |  | - symmetry in stance time; |
|  |  |  |  |  |  |  |  |  |  | - metabolic cost (PCI value); |
|  |  |  |  |  |  |  |  |  |  | - ability to walk on level ground; |
|  |  |  |  |  |  |  |  |  |  | - ability to negotiate slopes; |
|  |  |  |  |  |  |  |  |  |  | - ability to negotiate stairs; |
|  |  |  |  |  |  |  |  |  |  | - occurrence of stumbles; |
|  |  |  |  |  |  |  |  |  |  | - occurrence of falls; |
|  |  |  |  |  |  |  |  |  |  | - weight/comfort of prosthesis |
| Kahle 2008 [30] | 19 | 51.3 ± 19.4 (22-83) | within-subject | 10 | - trauma, PVD,   congenital, diabetes,   tumour;  - MFCL-2;  - MFCL-3;  - MFCL-4; | C-leg | weight- activated stance phase brake with pneumatic or friction swing phase; Mauch SNS; Total knee; 4-bar hydraulic swing; single axis friction | 90 days | - Prosthesis Evaluation   Questionnaire (PEQ);  - original questionnaire;  - walking speed tests;  - Montreal Rehabilitation   Performance Profile | - self-assessed satisfaction; |
|  |  |  |  |  |  |  |  |  |  | - self-assessed performance; |
|  |  |  |  |  |  |  |  |  |  | - number of stumbles; |
|  |  |  |  |  |  |  |  |  |  | - number of falls; |
|  |  |  |  |  |  |  |  |  |  | - self-selected walking speed (even ground); |
|  |  |  |  |  |  |  |  |  |  | - fastest possible walking speed (even ground); |
|  |  |  |  |  |  |  |  |  |  | - fastest possible walking speed (uneven   ground); |
|  |  |  |  |  |  |  |  |  |  | - ability to descend stairs (performance   composite score) |
| Kaufman 2008 [31] | 15 | 42 ± 9 (26-57) | within-subject | 10 | - trauma, tumour,   PVD, congenital;  - MFCL-3;  - MFCL-4 | C-leg | Mauch SNS; Catech; Black Max; Century 2000 | 10-39 weeks | - expired gas analysis during   treadmill walking;  - Borg rating of perceived exertion;  - doubly labelled water method;  - indirect calorimetry;  - Prosthesis Evaluation   Questionnaire (PEQ) | - VO_2_; |
|  |  |  |  |  |  |  |  |  |  | - VCO_2_; |
|  |  |  |  |  |  |  |  |  |  | - perceived effort of walking; |
|  |  |  |  |  |  |  |  |  |  | - physical activity-related energy expenditure   (PAEE); |
|  |  |  |  |  |  |  |  |  |  | - self-assessed performance; |
|  |  |  |  |  |  |  |  |  |  | - self-assessed satisfaction; |
| Berry 2009 [32] | 368 | 54.7 ± 15.6 (15-85) | within-subject | 10 | - trauma, tumour,   PVD, infection,   congenital, other;  - MFCL-3 | C-leg | variable cadence non-micro- processor- controlled | 6-9 months | - original questionnaire | - socket fit/comfort; |
|  |  |  |  |  |  |  |  |  |  | - confidence/security; |
|  |  |  |  |  |  |  |  |  |  | - gait/manoeuvrability; |
|  |  |  |  |  |  |  |  |  |  | - prosthesis attributes; |
|  |  |  |  |  |  |  |  |  |  | - physical effects of prosthesis; |
|  |  |  |  |  |  |  |  |  |  | - safety/negative attributes of prosthesis |
|  |  |  |  |  |  |  |  |  |  |  |
| Blumentritt 2009 [33] | 3 | 36.7 ± 10.1 (25-43) | within-subject | 10 | - trauma, tumour;  - MFCL-3;  - MFCL-4 | C-leg | Mauch SNS;  3R80 | 30 minutes | - video-based motion analysis;  - 2 force plates | - knee joint angle; |
|  |  |  |  |  |  |  |  |  |  | - sagittal knee moment; |
|  |  |  |  |  |  |  |  |  |  | - sagittal hip moment |
| Hafner 2009 [34] | 17 | 49.1 ± 16.4 (21-77) | within-subject | 11 | - trauma,   dysfunction,   malignancy,   infection, PVD;  - MFCL-2;  - MFCL-3 | C-leg | Seattle Mark V; Mauch; Teh Lin; Total knee; CaTech Hydraulic; OB active; Ultimate; 3R60; | 1-32 weeks | - amputee mobility predictor;  - 36-item short-form health survey   (SF-36);  - Stair Assessment Index (SAI);  - Hill Assessment Index (HAI);  - 73.2m outdoor obstacle course;  - distracted walking test;  - Prosthesis Evaluation   Questionnaire (PEQ);  - original 14-question PEQ   addendum | - basic functional mobility; |
|  |  |  |  |  |  |  |  |  |  | - self-reported general health (QoL); |
|  |  |  |  |  |  |  |  |  |  | - ability to negotiate stairs (functional   independence and technique); |
|  |  |  |  |  |  |  |  |  |  | - ability to negotiate slopes (functional   independence, technique, step length, speed); |
|  |  |  |  |  |  |  |  |  |  | - ability to negotiate uneven terrain (speed); |
|  |  |  |  |  |  |  |  |  |  | - cognitive demand of walking (test speed and   accuracy); |
|  |  |  |  |  |  |  |  |  |  | - self-assessed satisfaction; |
|  |  |  |  |  |  |  |  |  |  | - self-assessed performance; |
|  |  |  |  |  |  |  |  |  |  | - confidence; |
|  |  |  |  |  |  |  |  |  |  | - concentration; |
|  |  |  |  |  |  |  |  |  |  | - number of stumbles; |
|  |  |  |  |  |  |  |  |  |  | - number of falls |
| Mâaref 2010 [35] | 29 | 45 ± 14 (?-?) | 2 cohorts retro-spective | 7 | - trauma, tumour;  - non-vascular | C-leg | swing phase control | ≥1 month | - video-based motion analysis;  - 3 force plates | - walking speed; |
|  |  |  |  |  |  |  |  |  |  | - cadence; |
|  |  |  |  |  |  |  |  |  |  | - stride length; |
|  |  |  |  |  |  |  |  |  |  | - stance phase duration; |
|  |  |  |  |  |  |  |  |  |  | - full knee extension instant; |
|  |  |  |  |  |  |  |  |  |  | - latency period (LP) |
| Petersen 2010 [36] | 5 | 36.6 ±8.9 (26-48) | within-subject | 10 | - trauma, tumour;  - MFCL-3; - MFCL-4 | C-leg | 3R60 | 1 week | - video-based motion analysis;  - 2 force plates | - symmetry of step length; |
|  |  |  |  |  |  |  |  |  |  | - symmetry of stance phase duration; |
|  |  |  |  |  |  |  |  |  |  | - symmetry of vertical ground reaction |
|  |  |  |  |  |  |  |  |  |  | force (butterfly plot) |
| Highsmith 2011 [37] | 21 | 45.7 ± 14.9 (20-72) | 3 cohorts cross sectional | 11 | - trauma, tumour,   diabetes, PVD,   congenital;  - MFCL-2; MFCL-3;   MFCL-4 | C-leg; Power knee | Mauch SNS | ? | - video-based motion analysis;  - 2 force plates | - duration (stand to sit and sit to stand); |
|  |  |  |  |  |  |  |  |  |  | - vertical ground reaction force (vGRF); |
|  |  |  |  |  |  |  |  |  |  | - joint angle symmetry l/r (hip + knee); |
|  |  |  |  |  |  |  |  |  |  | - joint force symmetry l/r (hip + knee); |
|  |  |  |  |  |  |  |  |  |  | - joint moment symmetry l/r (hip + knee) |
| Theeven 2011 [38] | 30 | 59.1 ± (13.0) (?-?) | within-subject | 12 | - trauma, PVD,   tumour;  - MFCL-2 | C-leg; C-leg Compact | 3R80; 3R106; 3R60; 3R92; Acphapend; Ultimate; Total Knee; Mauch SNS; Graph-Lite; manual locking | 1 week | - Assessment of Daily Activities   Performance in Transfemoral   amputees test (ADAPT);  - original questionnaire | - ability to perform daily activities (performance   time);  - self-assessed ability to perform daily activities;  - knee preference |
| Burnfield 2012 [39] | 10 | 62 ± 11.3 (?-?) | within-subject | 10 | - MFCL-2 | C-leg Compact | 3R49; Hydraulic 4-bar; Total knee; 3R60; Mauch SNS; NOP4 | 3 months | - video-based motion analysis;  - intramuscular electromyography;  - insoles with compression closing   switches;  - timed up and go test (TUG);  - mobility subscale of the prosthesis   evaluation questionnaire (PEQ);  - activities-specific balance   confidence scale (ABC);  - Houghton scale | - walking velocity; |
|  |  |  |  |  |  |  |  |  |  | - stride length; |
|  |  |  |  |  |  |  |  |  |  | - cadence; |
|  |  |  |  |  |  |  |  |  |  | - stance duration; |
|  |  |  |  |  |  |  |  |  |  | - single limb support duration; |
|  |  |  |  |  |  |  |  |  |  | - moment of heel off; |
|  |  |  |  |  |  |  |  |  |  | - peak and mean EMG intensity; |
|  |  |  |  |  |  |  |  |  |  | - peak extension of hip in single limb support; |
|  |  |  |  |  |  |  |  |  |  | - peak knee flexion in weight acceptance and   single limb support; |
|  |  |  |  |  |  |  |  |  |  | - peak dorsiflexion of ankle in single limb   support; |
|  |  |  |  |  |  |  |  |  |  | - functional mobility; |
|  |  |  |  |  |  |  |  |  |  | - self-assessed performance; |
|  |  |  |  |  |  |  |  |  |  | - self-assessed satisfaction; |
|  |  |  |  |  |  |  |  |  |  | - balance confidence score; |
|  |  |  |  |  |  |  |  |  |  | - amount of prosthetic use; |
|  |  |  |  |  |  |  |  |  |  | - stability on different types of terrain |
| Kaufman 2012 [40] | 15 | 42 ± 9 (26-57) | within-subject | 9 | - trauma, tumour,   PVD, congenital;  - MFCL-3  - MFCL-4 | C-leg | Mauch SNS; Catech; Black Max; Century 2000 | 10-39 weeks | - video-based motion analysis;  - 4 force plates; | - joint angle symmetry index  - joint moment symmetry index |
| Schaarsmidt 2012 [41] | 5 | 42.6 ± 13.4 (24-61) | within-subject | 7 | - trauma; | C-leg | 3R80 | ? | - treadmill with built-in piezo   electric force transducers;  - four 3-axis force transducers   (ground); | - duration of double support phase; |
|  |  |  |  |  |  |  |  |  |  | - duration of single support phase; |
|  |  |  |  |  |  |  |  |  |  | - step time; |
|  |  |  |  |  |  |  |  |  |  | - contact time; |
|  |  |  |  |  |  |  |  |  |  | - horizontal and vertical ground reaction force   (GRF) maximum; |
|  |  |  |  |  |  |  |  |  |  | - vertical impulse; |
|  |  |  |  |  |  |  |  |  |  | - horizontal impulse; |
|  |  |  |  |  |  |  |  |  |  | - asymmetry factor of gait parameters |
| Theeven 2012 [42] | 30 | 59.1 ± (13.0) (?-?) | within-subject | 12 | - trauma, PVD,   tumour;  - MFCL-2 | C-leg; C-leg Compact | 3R80; 3R106; 3R60; 3R92; Acphapend; Ultimate; Total Knee; Mauch SNS; Graph-Lite; manual locking | 1 week | - prosthesis evaluation questionnaire   (PEQ);  - uniaxial accelerometer;  - activity diary; | - self-assessed satisfaction;  - self-assessed performance;  - number of daily bouts of activity;  - activity level;  - activity duration |
| Wong 2012 [43] | 1 | 53 | within- subject | 8 | - PVD;  - MFCL-3 | C-leg | ? | 12 months | - original questionnaire;  - activities-specific balance   confidence scale (ABC);  - BERG balance assessment;  - Timed up and go (TUG); | - ability to negotiate stairs (technique); |
|  |  |  |  |  |  |  |  |  |  | - participation in work and/or leisure activities; |
|  |  |  |  |  |  |  |  |  |  | - number of falls; |
|  |  |  |  |  |  |  |  |  |  | - balance confidence score; |
|  |  |  |  |  |  |  |  |  |  | - balance impairment score; |
|  |  |  |  |  |  |  |  |  |  | - functional mobility |

? = unknown; ESK = Endolite Stabilised Knee; IP = Intelligent Prosthesis; l/r = left/right; mech. knee = mechanically controlled prosthetic knee joint; MFCL = Medicare Functional Classification Level; MPK = microprocessor-controlled prosthetic knee joint; PSPC = pneumatic swing phase control; PVD = peripheral vascular disease; QoL = quality of life; sd = standard deviation; VT = Van Tulder score; VO_2_ = rate of oxygen consumption; VCO_2_ = rate of carbon dioxide production.
